# Efficacy and Safety of PSCK9 Inhibitors on Patients with Acute Coronary Syndrome: A Systematic Review and Meta-Analysis of Randomised Controlled Trials

**DOI:** 10.31083/j.rcm2503094

**Published:** 2024-03-07

**Authors:** Jiajing Zhao, Xinyu Tong, Jian Peng, Chuxin Lyu, Shu Lu

**Affiliations:** ^1^Graduate School, Nanjing University of Chinese Medicine, 210029 Nanjing, Jiangsu, China; ^2^Department of Cardiovascular, Wuxi Traditional Chinese Medicine Hospital, 214071 Wuxi, Jiangsu, China

**Keywords:** PSCK9I, acute coronary syndrome, systematic review, meta-analysis

## Abstract

**Background:**

PCSK9 MaB (Proprotein Convertase Subtilisin/Kexin Type 9 Inhibitor) may reduce the occurrence of major adverse cardiovascular events (MACEs) in patients diagnosed with acute coronary syndrome (ACS). In this meta-analysis, we conducted a thorough compilation of evidence from established clinical studies to evaluate PCSK9 MaB’s capacity to control blood lipid levels and prevent MACEs in ACS patients.

**Methods:**

We conducted searches on Pubmed, Embase, the Cochrane Library, and Web of Science to identify relevant articles. Data from ACS patients were extracted using a standardized format for aggregating data. We calculated the risk ratio (RR) for MACE and assessed changes in blood lipid parameters. All statistical analyses were performed using RevMan.

**Results:**

11 articles representing 5 trials were included in our systematic review and meta-analysis. When compared to a placebo, PCSK9 MaB significantly reduced the risk of MACEs (*$I^{2}$* = 0%, *p* = 0.63, RR [95% CI] = 0.88 [0.81, 0.97], *p* < 0.01) and the recurrence rate of ACS (*$I^{2}$* = 45%, *p* = 0.18, RR [95% CI] = 0.89 [0.83, 0.95], *p* < 0.01). Additionally, PCSK9 MaB notably reduced low-density lipoprotein cholesterol (LDL-C) levels (SMD [95% CI] = –2.12 [–2.32, –1.92], *p* < 0.01) and Apolipoprotein B (ApoB) levels (SMD [95% CI] = –1.83 [–2.48, –1.18], *p* < 0.01). Importantly, there were no significant differences in adverse reactions between the PCSK9 MaB group and the control group.

**Conclusions:**

PCSK9 MaB, whether used as a standalone treatment or in combination with other therapies, can effectively inhibit PCSK9. It substantially lowers key blood lipid parameters, including low-density lipoprotein (LDL), ApoB, and triglycerides, all without giving rise to notable safety concerns.

## 1. Introduction 

Acute coronary syndrome (ACS) encompasses unstable angina, non-ST-segment elevation myocardial infarction, and ST-segment elevation myocardial infarction [1]. Currently, ACS is highly prevalent among old people, with approximately 85% of ACS-related fatalities occurring in individuals aged 65 or older. It is worth noting that approximately 64% of ACS cases are attributed to the rupture of lipid plaques triggered by inflammation, followed by the formation of platelet-rich thrombosis [2]. In 2023, the European Society of Cardiology (ESC) recommended the combination of high-dose statins and ezetimibe as the standard approach to reducing blood lipid levels in ACS patients [3]. Patients with ACS exhibit a spectrum of clinical symptoms, ranging from mild manifestations like general fatigue to severe symptoms such as breathlessness and, in some cases, intense chest pain, which can pose a life-threatening risk in a short time frame. The pathophysiological changes in coronary syndrome share a common pattern: the transformation of coronary atherosclerotic plaques from a stable state to an unstable one, eventually culminating in plaque rupture and thrombosis [4]. Extensive clinical experience has demonstrated that the perturbation of blood lipid levels [5, 6] further exacerbates plaque instability, increasing the cardiac burden and progressively impeding vascular functions due to delayed blood flow metabolism and blockage of ion transport channels.

Proprotein convertase subtilisin/kexin type 9 inhibitor (PCSK9 MaB), belongs to a class of compounds designed to inhibit PCSK9, the ninth member of the Kexin-like proconvertase subtilisin family [7]. According to the clinical consensus statement endorsed by the Association for Acute Cardiovascular Care (ACVC) in collaboration with the European Association of Preventive Cardiology (EAPC) and the European Society of Cardiology (ESC) Working Group on Cardiovascular Pharmacotherapy, the use of PCSK9 MaB to treat ACS patients is both safe and feasible. This treatment approach aids in plaque stabilization and the prevention of plaque rupture [8].

The current lipid-lowering guidelines from the American College of Cardiology and the American Heart Association recommend adopting intermittent lipid-lowering therapy when an individual’s primary blood lipid levels consistently reach a safe target, such as maintaining low-density lipoprotein cholesterol (LDL-C) at 70 mg/dL through optimized treatment. This approach is especially relevant for patients at a very high risk of experiencing major adverse cardiovascular events (MACEs) [9]. Unsteady lipid-lowering therapy involves clinical treatment in a state of disturbance from external factors, with some interference left or removed, rather than returning to the original state. PCSK9 MaB is a suitable treatment option for these conditions. Such a recommendation is partially grounded in evidence from large clinical trials [8] demonstrating the clinical benefits of this therapy. Numerous prior experiments have confirmed that PCSK9 MaB effectively lowers blood lipid levels in 73.6% of ACS patients [10]. PCSK9 MaB reduces LDL-C, whether administered as monotherapy or in combination with statins. An added benefit of PCSK9 MaB is a 20% to 25% reduction in lipoprotein(a) concentrations [11]. However, approximately one-third of patients receiving PCSK9 MaB exhibit inadequate inhibition of coronary plaque development [12], leaving some patients vulnerable to adverse thrombotic events. The Further Cardiovascular Outcomes Research With PCSK9 monoclonal antibody (FOURIER) trial investigated patients with stable atherosclerotic cardiovascular disease and a previous myocardial infarction (MI). It found that PCSK9 MaB, particularly evolocumab, reduced MACEs in individuals with recent MIs (within 2 years) but did not yield the same effects in those with more remote MIs (beyond 2 years) [11]. As a result, PCSK9 MaB treatment should be considered for all ACS patients with dyslipidemia that remains uncontrolled despite statin therapy. While the effects of PCSK9 MaB have been demonstrated in some studies, it’s worth noting that this treatment can be relatively expensive and may raise concerns about potential MACEs, and its short-term clinical efficacy remains uncertain [13].

Statins have been a longstanding and well-established treatment in clinical practice, known for their clear lipid-lowering effects [3, 14]. In contrast, PCSK9 MaB is relatively new to clinical practice, and its efficacy requires validation through extensive data analysis. Consequently, a meta-analysis was undertaken, encompassing published randomized controlled trials (RCTs) and non-RCTs, to comprehensively assess the efficacy and safety of PCSK9 MaB for ACS.

## 2. Materials and Methods

This study adhered to the Preferred Reporting Items for Systematic reviews and Meta-Analyses (PRISMA) statement and was pre-registered on the PROSPERO platform with the registration number #CRD 42022372809 [15].

### 2.1 Literature Search

We conducted a thorough search of pertinent publications in the PUBMED, EMBASE, Cochrane Library, and Web of Science databases, encompassing the entire period from the inception of these databases up to October 2022. Our search terms were centered around “acute coronary syndrome” and “PCSK9 MaB”. The detailed search strategies and outcomes can be found in **Supplementary Table 1**.

No language restrictions were imposed during the search. We sought and identified all published studies, including both RCTs and non-RCTs that compared the effectiveness of PCSK9 MaB therapy with conventional or standard therapy for ACS patients. The precise keywords and subject headings employed in our search strategies can be found in **Supplementary Table 1**.

### 2.2 Study Inclusion Criteria

The inclusion criteria were outlined as follows: (1) Participants were adults, aged 18 years or older. (2) Participants had a history of any type of coronary syndrome, and the diagnostic criteria were explicitly defined in the article. (3) Participants had no mental disorders. (4) Eligible studies were either RCTs or non-RCTs (cohort studies) with independently extractable data. (5) The study population consisted of ACS patients who received PCSK9 MaB. (6) The primary endpoints of the studies encompassed blood lipid levels or clinical outcomes. Clinical ischemic outcomes were characterized as MACEs, which encompassed all-cause mortality, myocardial infarction, stroke, target vessel revascularization, or stent thrombosis.

### 2.3 Study Exclusion Criteria

The following studies were excluded based on the following criteria: (1) those that did not meet the inclusion criteria; (2) animal studies, case reports, conference papers, review articles, and abstracts; (3) studies lacking sufficient data; (4) studies for which access to the original articles and duplicate publications was not feasible; and (5) protocols or studies exclusively focused on genotype-guided strategies.

### 2.4 Data Extraction and Validity Assessment 

Two independent investigators conducted a meticulous review of all studies and collected pertinent data using a standardized table. In the event of any discrepancies in data extraction, a third reviewer was engaged in discussions and decision-making. The characteristics of each study were independently extracted, encompassing details such as study design, study population, ethnic background, patient count, average age, treatment approach, follow-up duration, and primary and secondary outcome measures. To assess the methodological quality of the included RCT studies, the Cochrane Collaboration Bias Assessment Tool (ROB 1.0), integrated into Review Manager (version 5.4.1, Northern Europe), was employed.

### 2.5 Statistical Analysis

We employed a random- or fixed-effects model to pool the data. The occurrence of MACEs or adverse events was compared between the genotype guidance group and the conventional group. The endpoint outcome was expressed as the risk ratio (RR) with a corresponding 95% confidence interval (CI). Simultaneously, the level of heterogeneity was quantified using the *$I^{2}$* statistic. An *$I^{2}$* value of ≤50% indicated low heterogeneity, while an *$I^{2}$* value exceeding 50% indicated high heterogeneity. We used the fixed-effects model when the *p*-value was ≥0.05 and *$I^{2}$* was ≤50%. Otherwise, the random-effects model was employed. In cases of heterogeneity, sensitivity analyses were performed by systematically excluding studies one by one to discern the potential impact of each study on the combined results. Funnel plots were employed to identify potential publication bias, but this approach was implemented only when the number of included studies exceeded 10, as publication bias tests based on a limited number of studies may yield unreliable results.

The reliability of the meta-analysis results was assessed by observing whether the pooled estimates remained consistent (based on the *p*-value). Statistical analyses were carried out using the Review Manager software (Review Manager (RevMan) [Computer program]. Version 5.4. The Cochrane Collaboration, 2020). All *p*-values were two-sided, and a *p*-value less than 0.05 was considered statistically significant.

## 3. Results

**Fig. 1.**
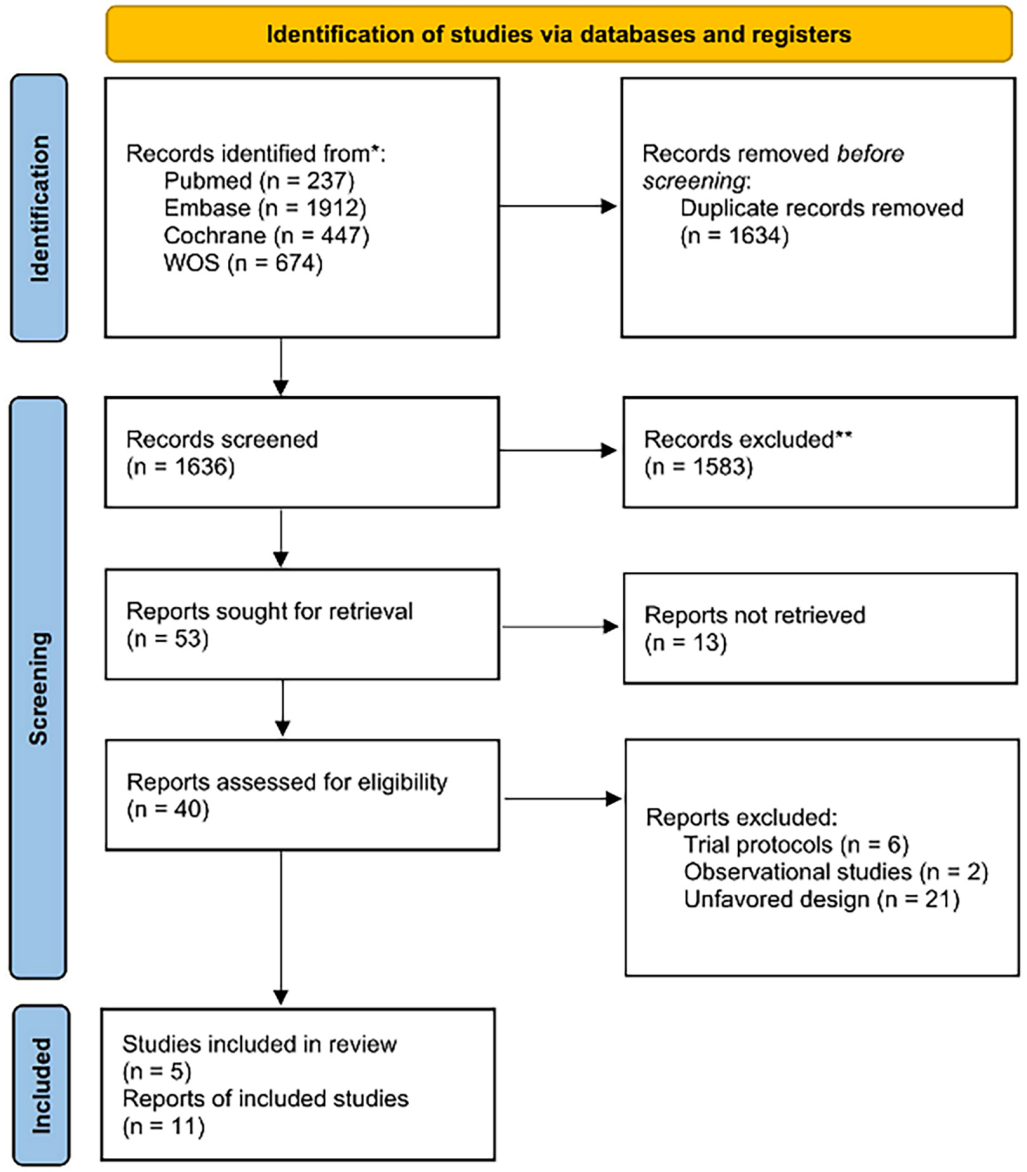
**Preferred Reporting Items for Systematic reviews and Meta-Analyses (PRISMA) Flowchart**.

Overall, this meta-analysis incorporated a total of 11 articles from 5 RCTs, with 7 of these articles pertaining to the ODISSEY trial, as depicted in Fig. 1. No publication bias tests were conducted for any of the outcome measures, given that the number of included studies was fewer than 10.

### 3.1 Basic Information

The patients had a mean age of 59.38 years, with a standard deviation of 10.6. Among the patients, 14,666 were male (74.6%), while 4983 were female (25.4%). The median follow-up duration ranged from 1.5 to 2.8 years. The PCSK9 MaB group included 9826 patients, and the conventional or standard group included 9823 patients. In the RCTs, no significant disparities in baseline characteristics were detected between the treatment and control groups, as indicated in Table 1 [4, 16, 17, 18, 19, 20, 21, 22, 23, 24, 25].

**Table 1.** **Characteristics of included reports**.

Author	Year	Region	Population	Age	Gender	PCSK9 MaB	Comparators	
				(mean, SD)	(female, male)			
Schwartz [16]	2018	Multiple	18,924	58.55, 9.35	4762, 14,162	Alirocumab, 70–150 mg, SC, biweekly	Placebo, SC, biweekly	ODISSEY Trial
Steg [17]	2019	Multiple	18,924	58.65, 9.64	4762, 14,162	Alirocumab, 70–150 mg, SC, biweekly	Placebo, SC, biweekly	
Goodman [18]	2019	Multiple	18,924	58.35, 10.34	4762, 14,162	Alirocumab, 70–150 mg, SC, biweekly	Placebo, SC, biweekly	
Damask [19]	2020	Multiple	11,953	58.6, 9.3	3039, 8914	Alirocumab, 70–150 mg, SC, biweekly	Placebo, SC, biweekly	
Hagström [20]	2022	Multiple	18,924	58.28, 10.18	4762, 14,162	Alirocumab, 70–150 mg, SC, biweekly	Placebo, SC, biweekly	
Schwartz [21]	2021	Multiple	18,924	58.19, 9.73	4762, 14,162	Alirocumab, 70–150 mg, SC, biweekly	Placebo, SC, biweekly	
Schwartz [22]	2021	Multiple	18,490	58.18, 9.64	4623, 13,867	Alirocumab, 70–150 mg, SC, biweekly	Placebo, SC, biweekly	
Koskinas [23]	2019	Switzerland	308	60.75, 11.36	57, 251	Evolocumab, 40 mg, everyday	Placebo, 40 mg, everyday	
Ako [4]	2019	Japan	182	61.16, 10.90	36, 146	Alirocumab, 70–150 mg, SC, biweekly	Standard of care	
Li [24]	2021	China	99	59.69, 10.33	35, 64	Evolocumab, 140 mg, SC, biweekly	Placebo, SC, biweekly	
						& Statins, 10 mg, SC, every night	& Statins, 10 mg, SC, every night	
Hao [25]	2022	China	136	62.21, 11.84	93, 43	Evolocumab, 140 mg, SC, biweekly	Placebo, SC, biweekly	

Abbreviation: PCSK9 MaB, proprotein convertase subtilisin/kexin type 9 inhibitor; SD, standard deviation; SC, subcutaneous injection. “Multi” means “multi-regional”. Notes: 1. Schwartz, Hagström, Steg, Goodman, Damask published 7 articles concerning the results of different populations based on ODISSEY. 2. Standard of care: It was defined as “stable dose statin therapy, with optional dose adjustment (within the range approved by health authority)”.

In the PCSK9 MaB group, a personalized approach was employed for blood lipid-lowering using PCSK9 MaBs. Alirocumab was utilized in 8 studies, while Evolocumab was used in 3 studies. Among the 3 studies involving Evolocumab, the control group received standard of care in 1 study and a placebo in 2 studies. Among the seven ODISSEY articles, Schwartz *et al*. [16, 21, 22] primarily focused on several aspects. Firstly, they examined the potential for the recurrence of ischemic cardiovascular events in patients with ACS. They aimed to determine whether Alirocumab could enhance cardiovascular outcomes in ACS patients who were already on high-intensity statins. Additionally, they investigated the impact of Alirocumab on LDL-C levels and lipoprotein(a) levels in ACS patients and explored the safety profile of Alirocumab in this patient group. Steg *et al*. [17] centered their study on evaluating the effect of Alirocumab on mortality in ACS patients, while Goodman *et al*. [18] emphasized the assessment of cardiovascular outcomes in ACS patients. Damask *et al*. [19] made a special effort to analyze the risk of MACEs. Hagström *et al*. [20] conducted a study that focused on the residual cardiovascular risk and Apolipoprotein B (ApoB) levels in ACS patients.

In terms of the study endpoints, 9 articles considered MACE as the primary endpoint, while 4 articles used any cardiovascular disease as the safety endpoint.

### 3.2 Quality Evaluation of Included Studies

**Fig. 2.**
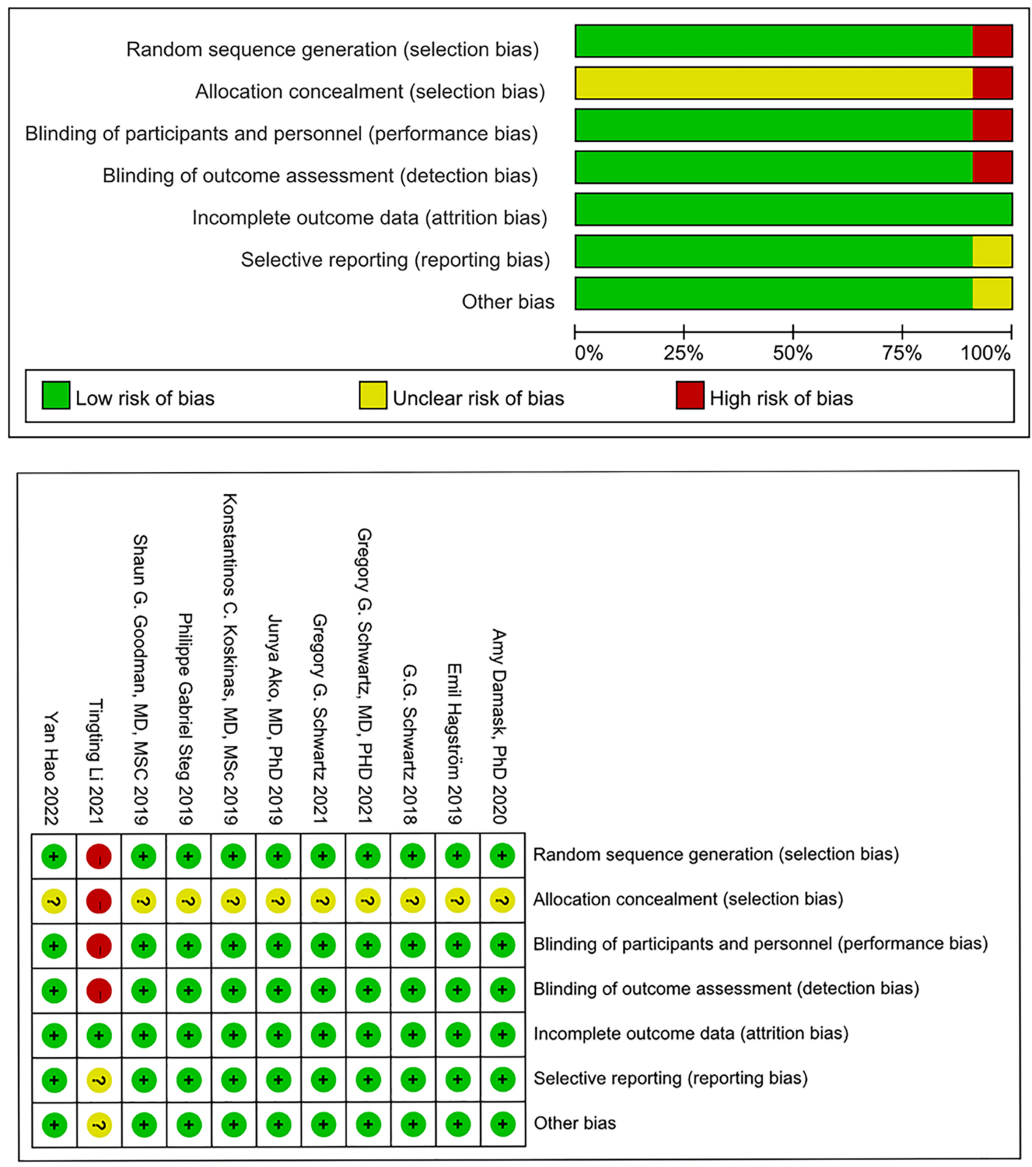
**Result of risk of bias assessment**.

As per the Cochrane Risk of Bias assessment tool, 10 articles, with the exception of Li *et al*. [24], utilized random sequence generation and allocation concealment methods. These studies were also conducted with double-blind designs. Given that all the RCTs included in the analysis provided complete data, the risk of bias in incomplete outcome data was deemed to be low. For the remaining potential sources of bias, the risk of bias in these studies was unclear. The risk of bias assessment results are presented in Fig. 2.

### 3.3 PXSK9 MaB Treatment and MACE 

**Fig. 3.**
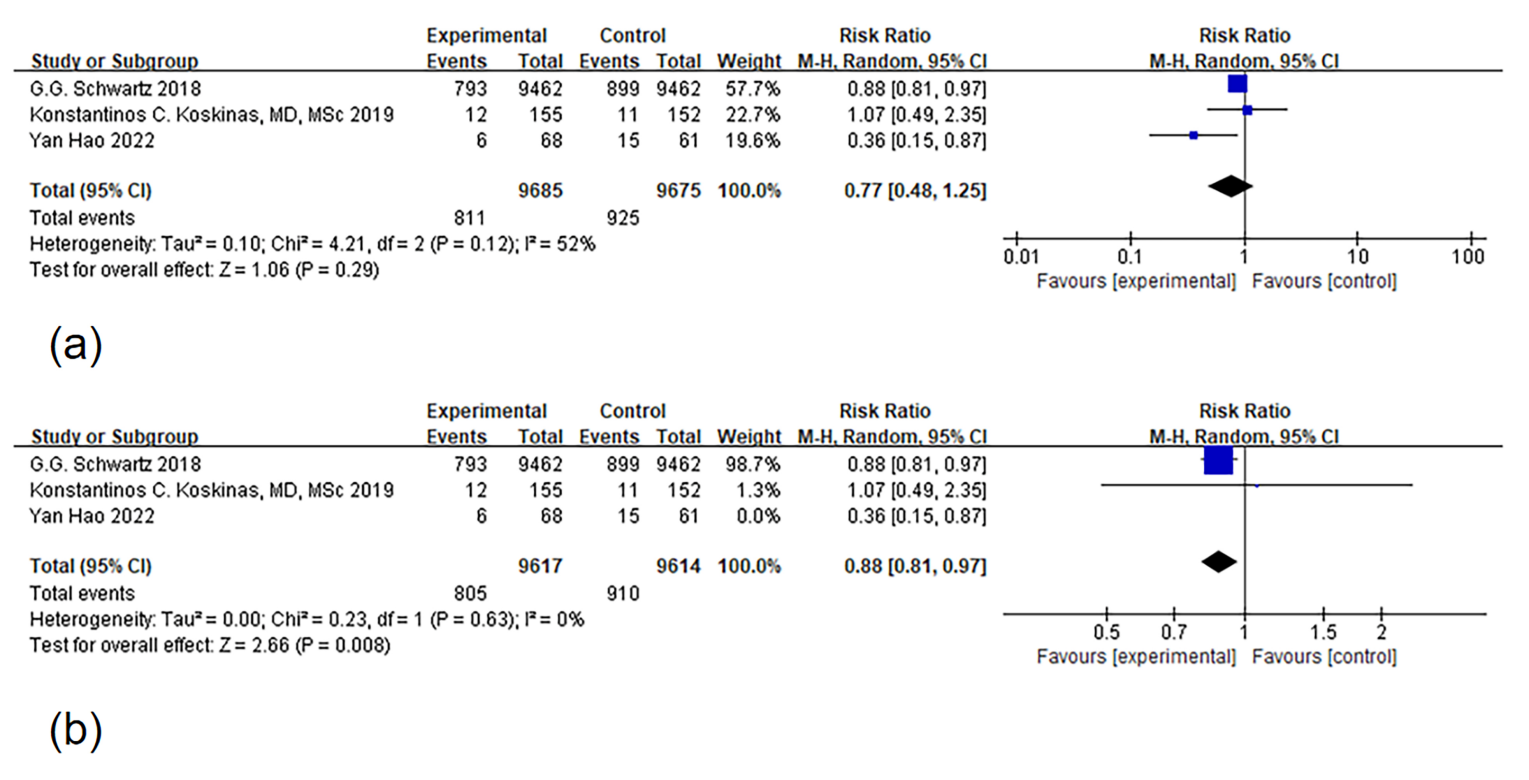
**Forest plot of major adverse cardiovascular event (MACE)**. Notes: (a) Forest map of MACE (b) Articles were systematically excluded one by one, and after this process, two articles remained for a thorough analysis of heterogeneity. The root of the heterogeneity was eventually traced back to the study conducted by Hao [25]. Upon exclusion of this particular study, the issue of heterogeneity ceased to be a factor.

In total, the ODISSEY studies encompassed 18,924 patients who were aged 40 years or older and had experienced ACS within 1 to 12 months prior to their randomization. MACEs occurred in 811 (8.25%) out of 9826 patients in the experimental group, while 925 (9.4%) out of 9823 patients in the control group experienced MACEs. Breaking down the individual endpoints, 786 patients (45.2%) suffered from all-cause death, 470 patients (27.1%) experienced myocardial infarctions, 394 patients (22.7%) had strokes, and 83 patients (4.8%) faced stent thrombosis. We conducted a meta-analysis for each MACE endpoint. Consequently, a total of 9 articles (comprising 7 ODISSEY articles and 2 RCTs) reported data related to the all-cause mortality. The results showed that PCSK9 MaB may reduce the risk of MACEs when compared to the control group. Notably, the 7 ODISSEY-related articles employed varying inclusion and exclusion criteria in the process of selecting participants, ultimately yielding diverse outcomes. Damask *et al*. [19] enrolled 11,953 ACS patients with pharmacogenomic data. Hagström *et al*. [20] concentrated on analyzing MACE in a cohort of 18,924 patients. Schwartz *et al*. [16] directed their attention towards investigating changes in lipid levels in the same group of 18,924 patients. Additionally, Schwartz *et al*. [22] initially screened 18,924 patients and ultimately included 18,490 individuals who met at least one of the following criteria: low-density lipoprotein (LDL) cholesterol level greater than or equal to 70 mg/dL (1.8 mmol/L), non-high-density lipoprotein (HDL) cholesterol level greater than or equal to 100 mg/dL (2.6 mmol/L), or apolipoprotein B level at least 80 mg/dL. Schwartz *et al*. [21] focused on analyzing MACE in all patients after 4 months of treatment. Steg *et al*. [17] focused on cardiovascular deaths, while Goodman *et al*. [18] categorized patients based on their coronary artery bypass grafting (CABG) status and specifically enrolled ACS patients without CABG (n = 16,896). After summarizing the diverse results, we found that, within the ODISSEY studies, PCSK9 MaB demonstrated the potential to reduce the incidence of MACE in patients (*p* < 0.01) (**Supplementary Figs. 1–4**). However, it is essential to note that the generalizability of this result may be limited. This is because these data were not derived from independent studies but to provide an overarching view of the trends in the findings across the ODISSEY-based studies. Subsequently, we conducted a meta-analysis on the data from the remaining two articles, as well as the ODISSEY-based article by Schwartz *et al*. [16]. Schwartz’s study is notable because it evaluated MACEs in all 18,924 enrolled participants without imposing any patient restrictions. In contrast, Hagström *et al*. [20] excluded patients with apoB levels less than 35 mg/dL, and all patients were randomly and equally grouped. The results of MACE risk derived from these studies held a more significant reference value compared to other ODISSEY studies. The analysis revealed substantial heterogeneity (*$I^{2}$* = 52%, *p* = 0.12) across the three articles. We systematically excluded articles one by one, eventually identifying that the source of heterogeneity was the study by Hao *et al*. [25]. Once this study was excluded, heterogeneity disappeared, and the meta-analysis showed that PCSK9 MaB, when compared to the control group, significantly reduced the risk of MACE with statistically significant differences (*$I^{2}$* = 0%, *p* = 0.63, RR [95% CI] = 0.88 [0.81, 0.97], *p* < 0.01), as depicted in Fig. 3 (Ref. [25]). Additionally, we conducted independent meta-analyses on the data from the other six ODISSEY-based articles and the remaining two non-ODISSEY articles. These results demonstrated that PCSK9 MaB could reduce the risk of MACEs to varying degrees (**Supplementary Table 2**).

### 3.4 PXSK9 MaB Treatment and Any ACS Recurrence 

**Fig. 4.**
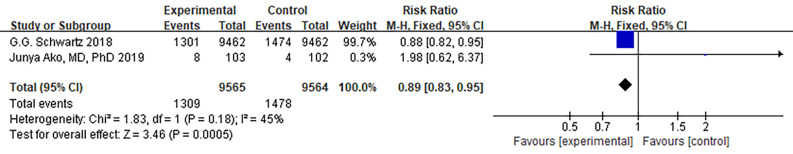
**Forest plot of acute coronary syndrome**. Abbreviation: 95% CI, 95% confidence interval.

ACS recurrence was reported in 3 ODISSEY-related articles, which employed differing inclusion and exclusion criteria for enrolling participants. Consequently, the outcome indicators varied across these studies. We meta-analyzed the diverse outcome data from these three articles and observed that PCSK9 MaB could reduce the recurrence rate of ACS (*p* < 0.01) within the context of the ODISSEY studies (Appendix). Damask *et al*. [19] collected DNA samples from a total of 12,118 patients out of the 18,924. Meanwhile, Goodman *et al*. [18] categorized patients based on their CABG status, specifically including those with CABG (n = 16,896), which resulted in differences in the total patient count. As a result, a meta-analysis was conducted using the data reported in the ODISSEY study by Schwartz *et al*. [21], which featured the most comprehensive patient dataset. In addition, data from one other study were included. This analysis indicated some degree of heterogeneity (*$I^{2}$* = 45%, *p* = 0.18, RR [95% CI] = 0.89 [0.83, 0.95], *p* < 0.01). However, the heterogeneity, while present, was not statistically significant. The findings showed that PCSK9 MaB significantly reduced the recurrence rate of ACS in patients, with statistical significance. To ensure the robustness of these results, a sensitivity analysis was conducted by sequentially excluding studies one by one, and no study omission affected the overall findings. Due to the relatively small number of included articles (less than 10), a publication bias test was not performed. The results are depicted in Fig. 4.

### 3.5 PXSK9 MaB Treatment and Blood Lipid Levels

#### 3.5.1 ApoB 

**Fig. 5.**
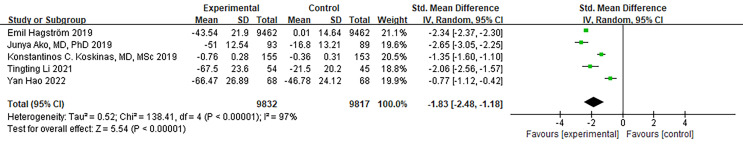
**Forest plot of ApoB**. Abbreviation: ApoB, Apolipoprotein B; 95% CI, 95% confidence interval.

Due to variations in the parameters measured across the five RCTs, the standardized mean difference (SMD) was chosen as the statistical measure. When comparing ApoB between the PCSK9 MaB group and the control group, we observed substantial heterogeneity (*$I^{2}$* = 97%, *p* < 0.01). However, it is important to note that although the heterogeneity was large, it was not statistically significant, suggesting that no significant source of heterogeneity was detected. Upon systematically excluding articles one by one, there was no major alteration in the level of heterogeneity. As a result, we employed a random-effects model for the analysis. The results demonstrated that ApoB levels (SMD [95% CI] = –1.83 [–2.48, –1.18], *p* < 0.01) were significantly lower in the PCSK9 MaB group than in the control group, with statistically significant differences (Fig. 5).

#### 3.5.2 LDL-C

**Fig. 6.**
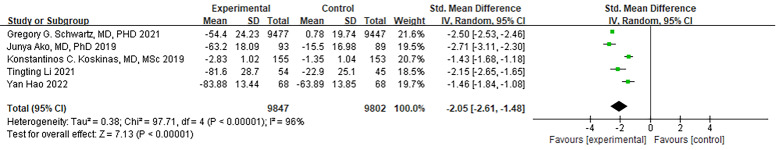
**Forest plot of LDL-C**. Abbreviation: LDL-C, Low density lipoprotein cholesterol; 95% CI, 95% confidence interval.

Both Hagström *et al*. [20] and Schwartz *et al*. [21] conducted ODISSEY-related studies that assessed and analyzed LDL-C values. However, Hagström *et al*. [20] excluded patients with apoB levels less than 35 mg/dL. Furthermore, LDL-C data was missing for 3 patients, which had an impact on the overall LDL-C results. Therefore, we performed a meta-analysis that included the ODISSEY study by Schwartz *et al*. [21] along with four other articles. The meta-analysis results indicated a significant decrease in LDL-C levels within the PCSK9 MaB group (SMD [95% CI] = –2.05 [–2.61, –1.48], *p* < 0.01). While there was notable heterogeneity (*$I^{2}$* = 96%, *p* < 0.01), this heterogeneity itself did not reach statistical significance. Given the relatively small number of articles included (only 5), a publication bias test was not conducted. The results are depicted in Fig. 6.

#### 3.5.3 Non–HDL-C

**Fig. 7.**
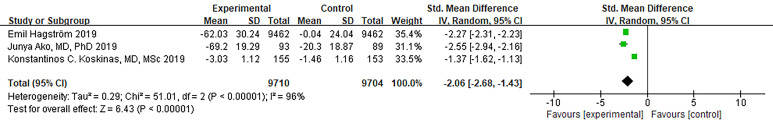
**Forest plot of Non–HDL-C**. Abbreviation: Non–HDL-C, non-high-density lipoprotein cholesterol; 95% CI, 95% confidence interval.

Three RCTs reported non-HDL-C endpoints. The analysis showed a significant level of heterogeneity (*$I^{2}$* = 96%, *p* < 0.01), but it is worth noting that this heterogeneity, although substantial, did not reach statistical significance. This suggests that no significant source of heterogeneity was identified. To ensure the robustness of the results, we systematically excluded articles one by one, and the results remained consistent. The meta-analysis revealed that PCSK9 MaB significantly reduced non-HDL-C levels when compared to the control group, with a statistically significant difference, as depicted in Fig. 7.

#### 3.5.4 Lipoprotein(a) 

**Fig. 8.**
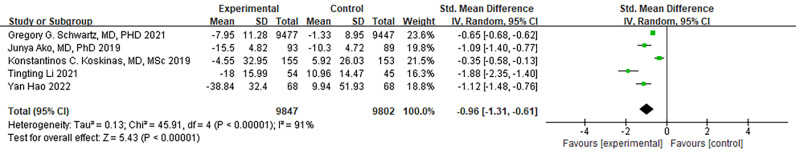
**Forest plot of Lipoprotein(a)**. Abbreviation: 95% CI, 95% confidence interval.

Both ODISSEY studies conducted by Hagström *et al*. [20] and Schwartz *et al*. [21] examined the levels of Lipoprotein(a). Notably, no drop-out patient data was observed in the entirety of Schwartz’s trial [21]. Consequently, these two studies, along with other four articles, were included in the meta-analysis. A random-effects model was used. While there was a substantial degree of heterogeneity, it did not reach statistical significance. To ensure the robustness of the results, we systematically excluded articles one by one, and no significant changes in the level of heterogeneity were detected. In summary, PCSK9 MaB significantly reduced the Lipoprotein(a) levels in ACS patients (*$I^{2}$* = 89%, *p* < 0.01; RR [95% CI] = –0.74 [–0.85, –0.63], *p* < 0.01), with a statistically significant difference, as depicted in Fig. 8.

#### 3.5.5 Triglycerides 

**Fig. 9.**
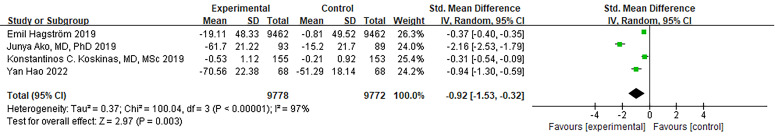
**Forest plot of Triglycerides**. Abbreviation: 95% CI, 95% confidence interval.

Four RCTs provided data on triglycerides. While there was a notable level of heterogeneity, it did not reach statistical significance (*$I^{2}$* = 97%, *p* = 0.04). A random-effects model was used. To ensure the reliability of the results, we conducted a sensitivity analysis by systematically excluding articles one by one. The analysis revealed that the level of heterogeneity remained relatively consistent. The meta-analysis demonstrated that PCSK9 MaB effectively reduced the levels of triglycerides in ACS patients when compared to the control group (SMD [95% CI] = –0.92 [–1.53, –0.32], *p* = 0.03), and this difference was statistically significant. These findings are visually represented in Fig. 9.

#### 3.5.6 HDL-C

**Fig. 10.**
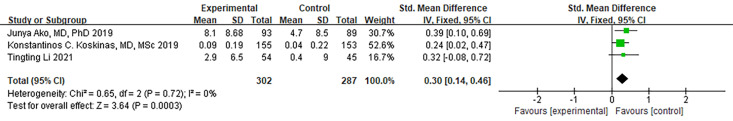
**Forest plot of HDL-C**. Abbreviation: HDL-C, high-density lipoprotein cholesterol; 95% CI, 95% confidence interval.

Three RCTs provided data on HDL-C. Notably, no significant heterogeneity was observed (*$I^{2}$* = 0%, *p* = 0.72). A fixed-effects model was used. The meta-analysis indicated that PCSK9 MaB effectively raised HDL-C levels when compared to the control group (SMD [95% CI] = 0.30 [0.14, 0.46], *p* = 0.0003), with a statistically significant difference. These results are visually presented in Fig. 10.

## 4. Discussion

To date, this study stands as the inaugural systematic review and meta-analysis aiming to assess the laboratory and clinical advantages of PCSK9 MaB to reduce lipid levels. Our findings may serve to foster a more holistic integration of PCSK9 MaB into clinical practice.

This systematic review and meta-analysis have unveiled the substantial benefits of personalized lipid-lowering treatment with PCSK9 MaB, notably in terms of lowering the risk of cardiovascular recurrence and MACEs in ACS patients when compared to conventional treatment. PCSK9 MaB is primarily synthesized in the endoplasmic reticulum. The process begins with the formation of a 75 kDa precursor, and this immature precursor comprises an N-terminal signal peptide sequence, a pre-domain, a catalytic domain, and a C-terminal domain rich in semiphotopine [18]. The binding of the pre-domain may play a role in inhibiting protease activity [26]. In ACS patients, cardiac overload and impaired blood flow contribute to MACE. Experiments have shown that lipoprotein lipase, a plasma-specific enzyme, acts as a catalyst in plasma [27], accelerating blood flow. After cleavage, the prestructure of PCSK9 MaB forms a non-covalent bond with the catalytic domain, creating a complex with mature fragments. It leaves the endoplasmic reticulum as a molecular chaperone and, following a series of modifications such as acetylation [28], is eventually secreted into the bloodstream. This process inhibits the activity of lipoprotein lipase, subsequently slowing down blood flow due to the loss of lipoprotein enzyme catalysis. PCSK9 MaB intervenes in the action mechanism of PCSK9, preserving protease activity. This blood protease can serve as a catalyst to restore normal blood flow speed and gradually enhance heart pumping function, thereby reducing the recurrence of cardiovascular events and the risk of MACE. This meta-analysis has demonstrated that PCSK9 MaB treatment effectively reduces blood lipid levels in ACS patients. PCSK9 MaB, serving as an enzyme that regulates nerve cell apoptosis, influences not only liver regeneration and nerve cell apoptosis but also the internal transformation of low-density lipoprotein receptor (LDLR). It reduces the number of LDLR in liver cells, thus impeding the clearance of LDL from the blood and leading to hypercholesterolemia [29]. Hypercholesterolemia is associated with liver-derived secreted protein that binds to the extracellular domain of LDLR and subsequently degrades LDLR within cells [30]. Studies have shown a significant positive correlation between blood PCSK9 levels and cholesterol, LDL, and triglyceride levels [31]. PCSK9 itself does not require the catalytic activity of kinases to impact LDLR transformation, and kinase activity does not guide or inhibit complex degradation [30]. As a serine protease, PCSK9 degrades LDLR, resulting in elevated blood LDL levels. Therefore, PCSK9 MaB, when used as monotherapy or in combination therapy, inhibits PCSK9 and effectively reduces LDL levels. For example, Evolocumab, an all-human IgG2 monoclonal antibody, serves as a PCSK9 inhibitor. It binds to PCSK9 and inhibits its interaction with low-density lipoprotein receptor and lipoprotein(a) receptor, thus preventing PCSK9-mediated degradation of these receptors [32]. Lipoprotein(a) is a low-density lipoprotein particle whose concentration is largely hereditary and is considered to have atherogenic, pro-inflammatory, and pro-thrombotic properties [16]. Additionally, PCSK9 MaB can reduce lipoprotein(a) concentrations by 20% to 25% [22]. Given the close connection between abnormal blood lipid parameters and a high risk of MACE, it is reasonable to expect that PCSK9 MaB can effectively lower the risk of MACE in these patients. Therefore, ACS patients receiving PCSK9 MaB may significantly benefit.

Plasma PCSK9 exists predominantly as a heterodimer (62 + 13 kDa), which is considered an active form due to its high affinity to LDL receptors (LDLR). A less active form of plasma PCSK9 with a lower molecular weight (with approximately two-fold reduced affinity to LDLR) can also be found [33]. In contrast, intracellular PCSK9 is only present in its proprotein form or as a preformed heterodimer. PCSK9 is involved both directly and indirectly in the process of atherosclerotic plaque formation [34]. PCSK9 MaB demonstrated a relatively safe profile with no significantly higher incidence of adverse events beyond cardiovascular events when compared to the control group. Previous studies have indicated that statin drugs can lead to abnormal liver function in some individuals with prolonged usage [25]. In this study, we explored this matter as well. Our findings revealed that, in contrast to a placebo, PCSK9 MaB had no greater impact on liver function. This suggests that PCSK9 MaB may offer an advantage over statins. In terms of the production of PCSK9, the PCSK9 zymogen is initially synthesized in the endoplasmic reticulum, and undergoes self-catalytic reactions either in the endoplasmic reticulum or golgiosome, leading to the cleavage and release of the propeptide. This process results in the formation of mature protease, which is immediately secreted into the bloodstream. PCSK9, through its regulation of LDLR, helps maintain the stability of plasma lipid levels, influences plasma cholesterol levels, modulates nerve cell apoptosis, and is associated with inflammatory responses [35]. As an inhibitor, PCSK9 MaB possesses various biological functions, including participation in nervous system development, regulation of nerve cell apoptosis, modulation of sodium channels, and influence on islet cell function. These functions may contribute to the restoration of impaired liver and kidney functions.

Currently, in addition to the studies mentioned above, there are several ongoing investigations on PCSK9 MaB. For example, the EVOLVE-MI study (NCT05284747) is assessing the clinical efficacy of PCSK9 MaB by monitoring the cumulative time to all-cause mortality in ACS patients over a 3.5-year period, as well as the changes in baseline LDL-C levels at 12 weeks and 52 weeks. Based on the findings of this meta-analysis, it is anticipated that all-cause mortality will be reduced, and there will be a significant improvement in baseline LDL-C levels. The VICTORION-INCEPTION trial (NCT04873934) is specifically focused on comparing the time it takes to achieve an LDL-C reduction <70 mg/dL when PCSK9 MaB is added to routine care, as opposed to routine care alone. PCSK9, functioning as a serine protease, degrades LDL receptors (LDLR), leading to increased blood LDL levels. PCSK9 MaB, whether used as a monotherapy or in combination, inhibits PCSK9’s action on LDLR, resulting in reduced LDL levels and a relatively faster improvement in LDL-C levels. In addition to MaB, attempts have been made to explore lipid-lowering therapies targeting the PCSK9 pathway. Based on the current research, small interfering RNA (siRNA) molecules have emerged as the next generation of drugs designed to counteract PCSK9. Inclisiran, an siRNA, is specifically designed to target PCSK9, thereby inhibiting the translation of PCSK9 messenger RNA (mRNA). This action results in reduced concentrations of the PCSK9 protein and, consequently, lower levels of LDL cholesterol [14]. Inclisiran functions by selectively silencing the translation of PCSK9 mRNA [36], resulting in a sustained reduction in LDL-C levels that can last up to 12 months [37, 38]. As a result, Inclisiran is regarded as a promising therapeutic approach worthy of further investigation. The findings from these studies offer valuable insights into future lipid-lowering therapies that target the PCSK9 pathway.

This study has several limitations worth noting. Firstly, certain studies exhibited substantial heterogeneity, and the source of this heterogeneity could not be effectively determined due to the limited number of included articles. Secondly, given that the majority of the studies were derived from the ODISSEY trials, the limited number of included studies, single clinical grouping, and lack of publication bias testing, further comparative investigations are required to gain a more comprehensive understanding of the causes of MACE risk.

## 5. Conclusions

When PCSK9 MaB is employed as a monotherapy or as part of combination therapy, ACS patients experience a reduced risk of cardiovascular recurrence events and MACEs. It is worth noting that patients at a high risk of ACS derive greater absolute benefits from PCSK9 MaB treatment. Additionally, PCSK9 MaB significantly lowers blood lipid parameters such as LDL, ApoB, and Triglycerides, without any discernible safety concerns. However, owing to the preponderance of the ODISSEY trials, the limited number of included studies, the single clinical grouping, and the absence of a publication bias analysis, further studies with more diverse clinical groupings and comparative analyses are warranted to investigate the underlying causes of MACE risk. Future meta-analyses should consider subdividing major adverse events and recurrent cardiovascular diseases within the ACS patient population. Moreover, a greater number of studies are needed to explore the specific impact of PCSK9 MaB on individual diseases.

## Data Availability

The original contributions presented in the study are included in the article/supplementary material, further inquiries can be directed to the corresponding author.

## Abbreviations

PCSK9, proprotein convertase subtilisin/kexin type 9 inhibitor; MACEs, major adverse cardiovascular events; ACS, acute coronary syndrome; RR, risk ratio; ACVC, Acute Cardiovascular Care; EAPC, European Association of Preventive Cardiology; ESC, European Society of Cardiology; LDL-C, low density lipoprotein cholesterol.

## Supplementary Material

Supplementary material associated with this article can be found, in the online version, at https://doi.org/10.31083/j.rcm2503094.
