# Supplementary material for: Efficacy and Safety of PSCK9 Inhibitors on Patients with Acute Coronary Syndrome: A Systematic Review and Meta-Analysis of Randomised Controlled Trials
Source: Rev Cardiovasc Med. 2024 Mar 7;25(3):94. doi: 10.31083/j.rcm2503094 (PMC11263862; doi:10.31083/j.rcm2503094)
Supplement: Supplementary file 1 [file 2153-8174-25-3-094-s1.zip › Supplementary materials.docx]

# Supplementary materials

Supplementary Table 1. Literature search strategy

**1.Pubmed**

| Search number | Query | Results |
| --- | --- | --- |
| #1 | ‘’Cardiovascular PCSK9 Inhibitors’’[Title/Abstract] OR ‘’Cardiovascular, PCSK9 Inhibitors’’[Title/Abstract] OR ‘’Inhibitors, Cardiovascular PCSK9’’[Title/Abstract] OR ‘’PCSK9 Inhibitors’’[Title/Abstract] OR ‘’PCSK9 Inhibitors Cardiovascular’’[Title/Abstract] OR ‘’PCSK9 Inhibitors, Cardiovascular‘’[Title/Abstract] | 2051 |
| #2 | PCSK9 Inhibitors[MeSH Terms] | 1052 |
| #3 | ‘’Myocardial Infarction’’[Title/Abstract] OR ‘’cardiac infarct’’[Title/Abstract] OR ‘’cardiac infarction’’[Title/Abstract] OR ‘’cardial infarct’’[Title/Abstract] OR ‘’Cardiovascular Stroke’’[Title/Abstract] OR ‘’Cardiovascular Strokes’’[Title/Abstract] OR ‘’heart attack’’[Title/Abstract] OR ‘’Heart Attacks’’[Title/Abstract] OR ‘’heart infarct’’[Title/Abstract] OR ‘’heart infarction’’[Title/Abstract] OR ‘’heart micro infarction’’[Title/Abstract] OR ‘’heart muscle infarction’’[Title/Abstract] OR ‘’Infarct, Myocardial’’[Title/Abstract] OR ‘’infarction, heart’’[Title/Abstract] OR ‘’Infarction, Myocardial’’[Title/Abstract] OR ‘’Infarctions, Myocardial’’[Title/Abstract] OR ‘’Infarcts, Myocardial’’[Title/Abstract] OR ‘’myocardial infarct’’[Title/Abstract] OR ‘’Myocardial Infarctions’’[Title/Abstract] OR ‘’Myocardial Infarcts’’[Title/Abstract] OR ‘’myocardium infarct’’[Title/Abstract] OR ‘’myocardium infarction’’[Title/Abstract] OR ‘’premonitory infarction sign’’[Title/Abstract] OR ‘’second heart attack’’[Title/Abstract] OR ‘’Stroke, Cardiovascular’’[Title/Abstract] OR ‘’Strokes, Cardiovascular’’[Title/Abstract] OR ‘’subendocardial infarction’’[Title/Abstract] OR ‘’transmural cardiac infarction’’[Title/Abstract] OR ‘’transmural heart infarction’’[Title/Abstract] OR ‘’transmural infarction, heart’’[Title/Abstract] | 234043 |
| #4 | Myocardial Infarction[MeSH Terms] | 190099 |
| #5 | (PCSK9 Inhibitors[MeSH Terms]) OR (‘’Cardiovascular PCSK9 Inhibitors’’[Title/Abstract] OR ‘’Cardiovascular, PCSK9 Inhibitors’’[Title/Abstract] OR ‘’Inhibitors, Cardiovascular PCSK9’’[Title/Abstract] OR ‘’PCSK9 Inhibitors’’[Title/Abstract] OR ‘’PCSK9 Inhibitors Cardiovascular’’[Title/Abstract] OR ‘’PCSK9 Inhibitors, Cardiovascular‘’[Title/Abstract]) | 2321 |
| #6 | ((PCSK9 Inhibitors[MeSH Terms]) OR (‘’Cardiovascular PCSK9 Inhibitors’’[Title/Abstract] OR ‘’Cardiovascular, PCSK9 Inhibitors’’[Title/Abstract] OR ‘’Inhibitors, Cardiovascular PCSK9’’[Title/Abstract] OR ‘’PCSK9 Inhibitors’’[Title/Abstract] OR ‘’PCSK9 Inhibitors Cardiovascular’’[Title/Abstract] OR ‘’PCSK9 Inhibitors, Cardiovascular‘’[Title/Abstract])) AND ((Myocardial Infarction[MeSH Terms]) OR (‘’Myocardial Infarction’’[Title/Abstract] OR ‘’cardiac infarct’’[Title/Abstract] OR ‘’cardiac infarction’’[Title/Abstract] OR ‘’cardial infarct’’[Title/Abstract] OR ‘’Cardiovascular Stroke’’[Title/Abstract] OR ‘’Cardiovascular Strokes’’[Title/Abstract] OR ‘’heart attack’’[Title/Abstract] OR ‘’Heart Attacks’’[Title/Abstract] OR ‘’heart infarct’’[Title/Abstract] OR ‘’heart infarction’’[Title/Abstract] OR ‘’heart micro infarction’’[Title/Abstract] OR ‘’heart muscle infarction’’[Title/Abstract] OR ‘’Infarct, Myocardial’’[Title/Abstract] OR ‘’infarction, heart’’[Title/Abstract] OR ‘’Infarction, Myocardial’’[Title/Abstract] OR ‘’Infarctions, Myocardial’’[Title/Abstract] OR ‘’Infarcts, Myocardial’’[Title/Abstract] OR ‘’myocardial infarct’’[Title/Abstract] OR ‘’Myocardial Infarctions’’[Title/Abstract] OR ‘’Myocardial Infarcts’’[Title/Abstract] OR ‘’myocardium infarct’’[Title/Abstract] OR ‘’myocardium infarction’’[Title/Abstract] OR ‘’premonitory infarction sign’’[Title/Abstract] OR ‘’second heart attack’’[Title/Abstract] OR ‘’Stroke, Cardiovascular’’[Title/Abstract] OR ‘’Strokes, Cardiovascular’’[Title/Abstract] OR ‘’subendocardial infarction’’[Title/Abstract] OR ‘’transmural cardiac infarction’’[Title/Abstract] OR ‘’transmural heart infarction’’[Title/Abstract] OR ‘’transmural infarction, heart’’[Title/Abstract])) | 230 |
| #7 | "Acute Coronary Syndrome"[Title/Abstract] OR "Acute Coronary Syndromes"[Title/Abstract] OR "Coronary Syndrome, Acute"[Title/Abstract] OR "Coronary Syndromes, Acute"[Title/Abstract] OR "Syndrome, Acute Coronary"[Title/Abstract] OR "Syndromes, Acute Coronary"[Title/Abstract] | 1222 |
| #8 | Acute Coronary Syndrome[MeSH Terms] | 684 |
| #9 | "Cardiovascular PCSK9 Inhibitors"[Title/Abstract] OR "Cardiovascular, PCSK9 Inhibitors"[Title/Abstract] OR "Inhibitors, Cardiovascular PCSK9"[Title/Abstract] OR "PCSK9 Inhibitors"[Title/Abstract] OR "PCSK9 Inhibitors Cardiovascular"[Title/Abstract] OR "PCSK9 Inhibitors, Cardiovascular"[Title/Abstract] | 82 |
| #10 | Cardiovascular PCSK9 Inhibitors[MeSH Terms] | 68 |
| #11 | (#7 OR #8) AND (#9 OR #10) | 6 |
| #12 | "alirocumab"[Title/Abstract] OR "aln 60212"[Title/Abstract] OR "aln pcssc"[Title/Abstract] OR "ALN PCSsc"[Title/Abstract] OR "aln60212"[Title/Abstract] OR "AMG 145"[Title/Abstract] OR "Cardiovascular PCSK9 Inhibitors"[Title/Abstract] OR "evolocumab"[Title/Abstract] OR "frovocimab"[Title/Abstract] OR "inclisiran"[Title/Abstract] OR "inclisiran sodium"[Title/Abstract] OR "kjx 839"[Title/Abstract] OR "kjx839"[Title/Abstract] OR "leqvio"[Title/Abstract] OR "ly 3015014"[Title/Abstract] OR "ly3015014"[Title/Abstract] OR "monoclonal antibody REGN727"[Title/Abstract] OR "PCSK9 Inhibitors"[Title/Abstract] OR "PCSK9 Inhibitors Cardiovascular"[Title/Abstract] OR "praluent"[Title/Abstract] OR "REGN 727"[Title/Abstract] OR "REGN727"[Title/Abstract] OR "REGN727 monoclonal antibody"[Title/Abstract] OR "repatha"[Title/Abstract] OR "SAR 236553"[Title/Abstract] OR "SAR236553"[Title/Abstract] | 122 |
| #13 | #12 AND (#7 OR #8) | 7 |

**2.Cochrane**

| Search number | Query | Results |
| --- | --- | --- |
| #1 | (‘Myocardial Infarction’ OR ‘cardiac infarct’ OR ‘cardiac infarction’ OR ‘cardial infarct’ OR ‘Cardiovascular Stroke’ OR ‘Cardiovascular Strokes’ OR ‘heart attack’ OR ‘Heart Attacks’ OR ‘heart infarct’ OR ‘heart infarction’ OR ‘heart micro infarction’ OR ‘heart muscle infarction’ OR ‘Infarct, Myocardial’ OR ‘infarction, heart’ OR ‘Infarction, Myocardial’ OR ‘Infarctions, Myocardial’ OR ‘Infarcts, Myocardial’ OR ‘myocardial infarct’ OR ‘Myocardial Infarctions’ OR ‘Myocardial Infarcts’ OR ‘myocardium infarct’ OR ‘myocardium infarction’ OR ‘premonitory infarction sign’ OR ‘second heart attack’ OR ‘Stroke, Cardiovascular’ OR ‘Strokes, Cardiovascular’ OR ‘subendocardial infarction’ OR ‘transmural cardiac infarction’ OR ‘transmural heart infarction’ OR ‘transmural infarction, heart’):ti,ab,kw | 45961 |
| #2 | MeSH descriptor: [Myocardial Infarction] explode all trees | 11858 |
| #3 | (‘alirocumab’ OR ‘aln 60212’ OR ‘aln pcssc’ OR ‘ALN PCSsc’ OR ‘aln60212’ OR ‘AMG 145’ OR ‘Cardiovascular PCSK9 Inhibitors’ OR ‘evolocumab’ OR ‘frovocimab’ OR ‘inclisiran’ OR ‘inclisiran sodium’ OR ‘kjx 839’ OR ‘kjx839’ OR ‘leqvio’ OR ‘ly 3015014’ OR ‘ly3015014’ OR ‘monoclonal antibody REGN727’ OR ‘PCSK9 Inhibitors’ OR ‘PCSK9 Inhibitors Cardiovascular’ OR ‘praluent’ OR ‘REGN 727’ OR ‘REGN727’ OR ‘REGN727 monoclonal antibody’ OR ‘repatha’ OR ‘SAR 236553’ OR ‘SAR236553’):ti,ab,kw | 1036 |
| #4 | (#1 OR #2) AND (#3) | 226 |
| #5 | (‘Cardiovascular PCSK9 Inhibitors’ OR ‘Cardiovascular, PCSK9 Inhibitors’ OR ‘Inhibitors, Cardiovascular PCSK9’ OR ‘PCSK9 Inhibitors’ OR ‘PCSK9 Inhibitors Cardiovascular’ OR ‘PCSK9 Inhibitors, Cardiovascular’):ti,ab,kw | 295 |
| #6 | MeSH descriptor: [PCSK9 Inhibitors] explode all trees | 89 |
| #7 | (‘Myocardial Infarction’ OR ‘cardiac infarct’ OR ‘cardiac infarction’ OR ‘cardial infarct’ OR ‘Cardiovascular Stroke’ OR ‘Cardiovascular Strokes’ OR ‘heart attack’ OR ‘Heart Attacks’ OR ‘heart infarct’ OR ‘heart infarction’ OR ‘heart micro infarction’ OR ‘heart muscle infarction’ OR ‘Infarct, Myocardial’ OR ‘infarction, heart’ OR ‘Infarction, Myocardial’ OR ‘Infarctions, Myocardial’ OR ‘Infarcts, Myocardial’ OR ‘myocardial infarct’ OR ‘Myocardial Infarctions’ OR ‘Myocardial Infarcts’ OR ‘myocardium infarct’ OR ‘myocardium infarction’ OR ‘premonitory infarction sign’ OR ‘second heart attack’ OR ‘Stroke, Cardiovascular’ OR ‘Strokes, Cardiovascular’ OR ‘subendocardial infarction’ OR ‘transmural cardiac infarction’ OR ‘transmural heart infarction’ OR ‘transmural infarction, heart’):ti,ab,kw | 45961 |
| #8 | MeSH descriptor: [Myocardial Infarction] explode all trees | 11858 |
| #9 | (#5 OR #6) AND (#7 OR #8) | 82 |
| #10 | (‘Acute Coronary Syndrome’ OR ‘Acute Coronary Syndromes’ OR ‘Coronary Syndrome, Acute’ OR ‘Coronary Syndromes, Acute’ OR ‘Syndrome, Acute Coronary’ OR ‘Syndromes, Acute Coronary’):ti,ab,kw | 8475 |
| #11 | MeSH descriptor: [Acute Coronary Syndrome] explode all trees | 2337 |
| #12 | (‘Cardiovascular PCSK9 Inhibitors’ OR ‘Cardiovascular, PCSK9 Inhibitors’ OR ‘Inhibitors, Cardiovascular PCSK9’ OR ‘PCSK9 Inhibitors’ OR ‘PCSK9 Inhibitors Cardiovascular’ OR ‘PCSK9 Inhibitors, Cardiovascular’):ti,ab,kw | 295 |
| #13 | MeSH descriptor: [PCSK9 Inhibitors] explode all trees | 89 |
| #14 | (#10 OR #11) AND (#12 OR #13) | 40 |
| #15 | (‘alirocumab’ OR ‘aln 60212’ OR ‘aln pcssc’ OR ‘ALN PCSsc’ OR ‘aln60212’ OR ‘AMG 145’ OR ‘Cardiovascular PCSK9 Inhibitors’ OR ‘evolocumab’ OR ‘frovocimab’ OR ‘inclisiran’ OR ‘inclisiran sodium’ OR ‘kjx 839’ OR ‘kjx839’ OR ‘leqvio’ OR ‘ly 3015014’ OR ‘ly3015014’ OR ‘monoclonal antibody REGN727’ OR ‘PCSK9 Inhibitors’ OR ‘PCSK9 Inhibitors Cardiovascular’ OR ‘praluent’ OR ‘REGN 727’ OR ‘REGN727’ OR ‘REGN727 monoclonal antibody’ OR ‘repatha’ OR ‘SAR 236553’ OR ‘SAR236553’):ti,ab,kw | 1036 |
| #16 | #15 AND (#10 OR #12) | 139 |

**3.Embase**

| Search number | Query | Results |
| --- | --- | --- |
| #1 | 'cardiovascular pcsk9 inhibitors':ti,ab,kw OR 'cardiovascular, pcsk9 inhibitors':ti,ab,kw OR 'inhibitors, cardiovascular pcsk9':ti,ab,kw OR 'pcsk9 inhibitors':ti,ab,kw OR 'pcsk9 inhibitors cardiovascular':ti,ab,kw OR 'pcsk9 inhibitors, cardiovascular':ti,ab,kw | 1977 |
| #2 | 'pcsk9 inhibitor'/exp OR 'pcsk9 inhibitor' | 4481 |
| #3 | 'myocardial infarction':ti,ab,kw OR 'cardiac infarct':ti,ab,kw OR 'cardiac infarction':ti,ab,kw OR 'cardial infarct':ti,ab,kw OR 'cardiovascular stroke':ti,ab,kw OR 'cardiovascular strokes':ti,ab,kw OR 'heart attack':ti,ab,kw OR 'heart attacks':ti,ab,kw OR 'heart infarct':ti,ab,kw OR 'heart infarction':ti,ab,kw OR 'heart micro infarction':ti,ab,kw OR 'heart muscle infarction':ti,ab,kw OR 'infarct, myocardial':ti,ab,kw OR 'infarction, heart':ti,ab,kw OR 'infarction, myocardial':ti,ab,kw OR 'infarctions, myocardial':ti,ab,kw OR 'infarcts, myocardial':ti,ab,kw OR 'myocardial infarct':ti,ab,kw OR 'myocardial infarctions':ti,ab,kw OR 'myocardial infarcts':ti,ab,kw OR 'myocardium infarct':ti,ab,kw OR 'myocardium infarction':ti,ab,kw OR 'premonitory infarction sign':ti,ab,kw OR 'second heart attack':ti,ab,kw OR 'stroke, cardiovascular':ti,ab,kw OR 'strokes, cardiovascular':ti,ab,kw OR 'subendocardial infarction':ti,ab,kw OR 'transmural cardiac infarction':ti,ab,kw OR 'transmural heart infarction':ti,ab,kw OR 'transmural infarction, heart':ti,ab,kw | 339173 |
| #4 | 'heart infarction'/exp | 446443 |
| #5 | (#1 OR #2) AND (#3 OR #4) | 959 |
| #6 | 'alirocumab':ti,ab,kw OR 'aln 60212':ti,ab,kw OR 'aln pcssc':ti,ab,kw OR 'aln60212':ti,ab,kw OR 'amg 145':ti,ab,kw OR 'cardiovascular pcsk9 inhibitors':ti,ab,kw OR 'evolocumab':ti,ab,kw OR 'frovocimab':ti,ab,kw OR 'inclisiran':ti,ab,kw OR 'inclisiran sodium':ti,ab,kw OR 'kjx 839':ti,ab,kw OR 'kjx839':ti,ab,kw OR 'leqvio':ti,ab,kw OR 'ly 3015014':ti,ab,kw OR 'ly3015014':ti,ab,kw OR 'monoclonal antibody regn727':ti,ab,kw OR 'pcsk9 inhibitors':ti,ab,kw OR 'pcsk9 inhibitors cardiovascular':ti,ab,kw OR 'praluent':ti,ab,kw OR 'regn 727':ti,ab,kw OR 'regn727':ti,ab,kw OR 'regn727 monoclonal antibody':ti,ab,kw OR 'repatha':ti,ab,kw OR 'sar 236553':ti,ab,kw OR 'sar236553':ti,ab,kw | 3623 |
| #7 | 'myocardial infarction':ti,ab,kw OR 'cardiac infarct':ti,ab,kw OR 'cardiac infarction':ti,ab,kw OR 'cardial infarct':ti,ab,kw OR 'cardiovascular stroke':ti,ab,kw OR 'cardiovascular strokes':ti,ab,kw OR 'heart attack':ti,ab,kw OR 'heart attacks':ti,ab,kw OR 'heart infarct':ti,ab,kw OR 'heart infarction':ti,ab,kw OR 'heart micro infarction':ti,ab,kw OR 'heart muscle infarction':ti,ab,kw OR 'infarct, myocardial':ti,ab,kw OR 'infarction, heart':ti,ab,kw OR 'infarction, myocardial':ti,ab,kw OR 'infarctions, myocardial':ti,ab,kw OR 'infarcts, myocardial':ti,ab,kw OR 'myocardial infarct':ti,ab,kw OR 'myocardial infarctions':ti,ab,kw OR 'myocardial infarcts':ti,ab,kw OR 'myocardium infarct':ti,ab,kw OR 'myocardium infarction':ti,ab,kw OR 'premonitory infarction sign':ti,ab,kw OR 'second heart attack':ti,ab,kw OR 'stroke, cardiovascular':ti,ab,kw OR 'strokes, cardiovascular':ti,ab,kw OR 'subendocardial infarction':ti,ab,kw OR 'transmural cardiac infarction':ti,ab,kw OR 'transmural heart infarction':ti,ab,kw OR 'transmural infarction, heart':ti,ab,kw | 339279 |
| #8 | 'heart infarction'/exp | 446570 |
| #9 | #6 AND (#7 OR #8) | 587 |
| #10 | 'acute coronary syndrome':ti,ab,kw OR 'acute coronary syndromes':ti,ab,kw OR 'coronary syndrome, acute':ti,ab,kw OR 'coronary syndromes, acute':ti,ab,kw OR 'syndrome, acute coronary':ti,ab,kw OR 'syndromes, acute coronary':ti,ab,kw | 68075 |
| #11 | 'acute coronary syndrome'/exp | 70632 |
| #12 | 'cardiovascular pcsk9 inhibitors':ti,ab,kw OR 'cardiovascular, pcsk9 inhibitors':ti,ab,kw OR 'inhibitors, cardiovascular pcsk9':ti,ab,kw OR 'pcsk9 inhibitors':ti,ab,kw OR 'pcsk9 inhibitors cardiovascular':ti,ab,kw OR 'pcsk9 inhibitors, cardiovascular':ti,ab,kw | 1985 |
| #13 | 'cardiovascular':ti,ab,kw OR 'pcsk9 inhibitors':ti,ab,kw | 1886 |
| #14 | (#10 OR #11) AND (#12 OR #13) | 223 |
| #15 | 'alirocumab':ti,ab,kw OR 'aln 60212':ti,ab,kw OR 'aln pcssc':ti,ab,kw OR 'aln60212':ti,ab,kw OR 'amg 145':ti,ab,kw OR 'cardiovascular pcsk9 inhibitors':ti,ab,kw OR 'evolocumab':ti,ab,kw OR 'frovocimab':ti,ab,kw OR 'inclisiran':ti,ab,kw OR 'inclisiran sodium':ti,ab,kw OR 'kjx 839':ti,ab,kw OR 'kjx839':ti,ab,kw OR 'leqvio':ti,ab,kw OR 'ly 3015014':ti,ab,kw OR 'ly3015014':ti,ab,kw OR 'monoclonal antibody regn727':ti,ab,kw OR 'pcsk9 inhibitors':ti,ab,kw OR 'pcsk9 inhibitors cardiovascular':ti,ab,kw OR 'praluent':ti,ab,kw OR 'regn 727':ti,ab,kw OR 'regn727':ti,ab,kw OR 'regn727 monoclonal antibody':ti,ab,kw OR 'repatha':ti,ab,kw OR 'sar 236553':ti,ab,kw OR 'sar236553':ti,ab,kw | 3641 |
| #16 | #15 AND (#10 OR #11) | 366 |

**4.Web of science**

| Search number | Query | Results |
| --- | --- | --- |
| #1 | (TI=((Myocardial Infarction) OR (cardiac infarct) OR (cardiac infarction) OR (cardial infarct) OR (Cardiovascular Stroke) OR (Cardiovascular Strokes) OR (heart attack) OR (Heart Attacks) OR (heart infarct) OR (heart infarction) OR (heart micro infarction) OR (heart muscle infarction) OR (Infarct, Myocardial) OR (infarction, heart) OR (Infarction, Myocardial) OR (Infarctions, Myocardial) OR (Infarcts, Myocardial) OR (myocardial infarct) OR (Myocardial Infarctions) OR (Myocardial Infarcts) OR (myocardium infarct) OR (myocardium infarction) OR (premonitory infarction sign) OR (second heart attack) OR (Stroke, Cardiovascular) OR (Strokes, Cardiovascular) OR (subendocardial infarction) OR (transmural cardiac infarction) OR (transmural heart infarction) OR (transmural infarction, heart)) OR AB=((Myocardial Infarction) OR (cardiac infarct) OR (cardiac infarction) OR (cardial infarct) OR (Cardiovascular Stroke) OR (Cardiovascular Strokes) OR (heart attack) OR (Heart Attacks) OR (heart infarct) OR (heart infarction) OR (heart micro infarction) OR (heart muscle infarction) OR (Infarct, Myocardial) OR (infarction, heart) OR (Infarction, Myocardial) OR (Infarctions, Myocardial) OR (Infarcts, Myocardial) OR (myocardial infarct) OR (Myocardial Infarctions) OR (Myocardial Infarcts) OR (myocardium infarct) OR (myocardium infarction) OR (premonitory infarction sign) OR (second heart attack) OR (Stroke, Cardiovascular) OR (Strokes, Cardiovascular) OR (subendocardial infarction) OR (transmural cardiac infarction) OR (transmural heart infarction) OR (transmural infarction, heart)) OR AK=((Myocardial Infarction) OR (cardiac infarct) OR (cardiac infarction) OR (cardial infarct) OR (Cardiovascular Stroke) OR (Cardiovascular Strokes) OR (heart attack) OR (Heart Attacks) OR (heart infarct) OR (heart infarction) OR (heart micro infarction) OR (heart muscle infarction) OR (Infarct, Myocardial) OR (infarction, heart) OR (Infarction, Myocardial) OR (Infarctions, Myocardial) OR (Infarcts, Myocardial) OR (myocardial infarct) OR (Myocardial Infarctions) OR (Myocardial Infarcts) OR (myocardium infarct) OR (myocardium infarction) OR (premonitory infarction sign) OR (second heart attack) OR (Stroke, Cardiovascular) OR (Strokes, Cardiovascular) OR (subendocardial infarction) OR (transmural cardiac infarction) OR (transmural heart infarction) OR (transmural infarction, heart))) AND (TI=((Cardiovascular PCSK9 Inhibitors) OR (Cardiovascular, PCSK9 Inhibitors) OR (Inhibitors, Cardiovascular PCSK9) OR (PCSK9 Inhibitors) OR (PCSK9 Inhibitors Cardiovascular) OR (PCSK9 Inhibitors, Cardiovascular)) OR AB=((Cardiovascular PCSK9 Inhibitors) OR (Cardiovascular, PCSK9 Inhibitors) OR (Inhibitors, Cardiovascular PCSK9) OR (PCSK9 Inhibitors) OR (PCSK9 Inhibitors Cardiovascular) OR (PCSK9 Inhibitors, Cardiovascular)) OR AK=((Cardiovascular PCSK9 Inhibitors) OR (Cardiovascular, PCSK9 Inhibitors) OR (Inhibitors, Cardiovascular PCSK9) OR (PCSK9 Inhibitors) OR (PCSK9 Inhibitors Cardiovascular) OR (PCSK9 Inhibitors, Cardiovascular))) | 198 |
| #2 | (TI=((alirocumab) OR (aln 60212) OR (aln pcssc) OR (ALN PCSsc) OR (aln60212) OR (AMG 145) OR (Cardiovascular PCSK9 Inhibitors) OR (evolocumab) OR (frovocimab) OR (inclisiran) OR (inclisiran sodium) OR (kjx 839) OR (kjx839) OR (leqvio) OR (ly 3015014) OR (ly3015014) OR (monoclonal antibody REGN727) OR (PCSK9 Inhibitors) OR (PCSK9 Inhibitors Cardiovascular) OR (praluent) OR (REGN 727) OR (REGN727) OR (REGN727 monoclonal antibody) OR (repatha) OR (SAR 236553) OR (SAR236553)) OR AB=((alirocumab) OR (aln 60212) OR (aln pcssc) OR (ALN PCSsc) OR (aln60212) OR (AMG 145) OR (Cardiovascular PCSK9 Inhibitors) OR (evolocumab) OR (frovocimab) OR (inclisiran) OR (inclisiran sodium) OR (kjx 839) OR (kjx839) OR (leqvio) OR (ly 3015014) OR (ly3015014) OR (monoclonal antibody REGN727) OR (PCSK9 Inhibitors) OR (PCSK9 Inhibitors Cardiovascular) OR (praluent) OR (REGN 727) OR (REGN727) OR (REGN727 monoclonal antibody) OR (repatha) OR (SAR 236553) OR (SAR236553)) OR AK=((alirocumab) OR (aln 60212) OR (aln pcssc) OR (ALN PCSsc) OR (aln60212) OR (AMG 145) OR (Cardiovascular PCSK9 Inhibitors) OR (evolocumab) OR (frovocimab) OR (inclisiran) OR (inclisiran sodium) OR (kjx 839) OR (kjx839) OR (leqvio) OR (ly 3015014) OR (ly3015014) OR (monoclonal antibody REGN727) OR (PCSK9 Inhibitors) OR (PCSK9 Inhibitors Cardiovascular) OR (praluent) OR (REGN 727) OR (REGN727) OR (REGN727 monoclonal antibody) OR (repatha) OR (SAR 236553) OR (SAR236553))) AND (TI=((Myocardial Infarction) OR (cardiac infarct) OR (cardiac infarction) OR (cardial infarct) OR (Cardiovascular Stroke) OR (Cardiovascular Strokes) OR (heart attack) OR (Heart Attacks) OR (heart infarct) OR (heart infarction) OR (heart micro infarction) OR (heart muscle infarction) OR (Infarct, Myocardial) OR (infarction, heart) OR (Infarction, Myocardial) OR (Infarctions, Myocardial) OR (Infarcts, Myocardial) OR (myocardial infarct) OR (Myocardial Infarctions) OR (Myocardial Infarcts) OR (myocardium infarct) OR (myocardium infarction) OR (premonitory infarction sign) OR (second heart attack) OR (Stroke, Cardiovascular) OR (Strokes, Cardiovascular) OR (subendocardial infarction) OR (transmural cardiac infarction) OR (transmural heart infarction) OR (transmural infarction, heart)) OR AB=((Myocardial Infarction) OR (cardiac infarct) OR (cardiac infarction) OR (cardial infarct) OR (Cardiovascular Stroke) OR (Cardiovascular Strokes) OR (heart attack) OR (Heart Attacks) OR (heart infarct) OR (heart infarction) OR (heart micro infarction) OR (heart muscle infarction) OR (Infarct, Myocardial) OR (infarction, heart) OR (Infarction, Myocardial) OR (Infarctions, Myocardial) OR (Infarcts, Myocardial) OR (myocardial infarct) OR (Myocardial Infarctions) OR (Myocardial Infarcts) OR (myocardium infarct) OR (myocardium infarction) OR (premonitory infarction sign) OR (second heart attack) OR (Stroke, Cardiovascular) OR (Strokes, Cardiovascular) OR (subendocardial infarction) OR (transmural cardiac infarction) OR (transmural heart infarction) OR (transmural infarction, heart)) OR AK=((Myocardial Infarction) OR (cardiac infarct) OR (cardiac infarction) OR (cardial infarct) OR (Cardiovascular Stroke) OR (Cardiovascular Strokes) OR (heart attack) OR (Heart Attacks) OR (heart infarct) OR (heart infarction) OR (heart micro infarction) OR (heart muscle infarction) OR (Infarct, Myocardial) OR (infarction, heart) OR (Infarction, Myocardial) OR (Infarctions, Myocardial) OR (Infarcts, Myocardial) OR (myocardial infarct) OR (Myocardial Infarctions) OR (Myocardial Infarcts) OR (myocardium infarct) OR (myocardium infarction) OR (premonitory infarction sign) OR (second heart attack) OR (Stroke, Cardiovascular) OR (Strokes, Cardiovascular) OR (subendocardial infarction) OR (transmural cardiac infarction) OR (transmural heart infarction) OR (transmural infarction, heart))) | 295 |
| #3 | (TI=((Acute Coronary Syndrome) OR (Acute Coronary Syndromes) OR (Coronary Syndrome,  Acute) OR (Coronary Syndromes, Acute) OR (Syndrome, Acute Coronary) OR (Syndromes,  Acute Coronary)) OR AB=((Acute Coronary Syndrome) OR (Acute Coronary Syndromes) OR  (Coronary Syndrome, Acute) OR (Coronary Syndromes, Acute) OR (Syndrome, Acute  Coronary) OR (Syndromes, Acute Coronary)) OR AK=((Acute Coronary Syndrome) OR (Acute  Coronary Syndromes) OR (Coronary Syndrome, Acute) OR (Coronary Syndromes, Acute) OR  (Syndrome, Acute Coronary) OR (Syndromes, Acute Coronary))) AND (TI=((alirocumab) OR (aln  60212) OR (aln pcssc) OR (ALN PCSsc) OR (aln60212) OR (AMG 145) OR (Cardiovascular  PCSK9 Inhibitors) OR (evolocumab) OR (frovocimab) OR (inclisiran) OR (inclisiran sodium) OR  (kjx 839) OR (kjx839) OR (leqvio) OR (ly 3015014) OR (ly3015014) OR (monoclonal antibody  REGN727) OR (PCSK9 Inhibitors) OR (PCSK9 Inhibitors Cardiovascular) OR (praluent) OR  (REGN 727) OR (REGN727) OR (REGN727 monoclonal antibody) OR (repatha) OR (SAR  236553) OR (SAR236553)) OR AB=((alirocumab) OR (aln 60212) OR (aln pcssc) OR (ALN  PCSsc) OR (aln60212) OR (AMG 145) OR (Cardiovascular PCSK9 Inhibitors) OR (evolocumab)  OR (frovocimab) OR (inclisiran) OR (inclisiran sodium) OR (kjx 839) OR (kjx839) OR (leqvio) OR(ly 3015014) OR (ly3015014) OR (monoclonal antibody REGN727) OR (PCSK9 Inhibitors) OR(PCSK9 Inhibitors Cardiovascular) OR (praluent) OR (REGN 727) OR (REGN727) OR  (REGN727 monoclonal antibody) OR (repatha) OR (SAR 236553) OR (SAR236553)) OR  AK=((alirocumab) OR (aln 60212) OR (aln pcssc) OR (ALN PCSsc) OR (aln60212) OR (AMG  145) OR (Cardiovascular PCSK9 Inhibitors) OR (evolocumab) OR (frovocimab) OR (inclisiran)  OR (inclisiran sodium) OR (kjx 839) OR (kjx839) OR (leqvio) OR (ly 3015014) OR (ly3015014)  OR (monoclonal antibody REGN727) OR (PCSK9 Inhibitors) OR (PCSK9 Inhibitors  Cardiovascular) OR (praluent) OR (REGN 727) OR (REGN727) | 181 |

Supplementary Table 2. Meta analysis of different results of ODESSEY trials

|  | Heterogeneity | Methods | RR[95%CI] | *p* of effect |
| --- | --- | --- | --- | --- |
| ODESSEY | *I^2^* = 51%, *p* = 0.06 | REM | 0.88 [0.82, 0.94] | < 0.01 |
| MA1 Damask, 2020 | *I^2^* = 49%, *p* = 0.14 | FEM | 0.83 [0.75, 0.92] | < 0.01 |
| MA2 Hagström 2019 | *I^2^* = 49%, *p* = 0.14 | FEM | 0.83 [0.72, 0.96] | 0.01 |
| MA3 Schwartz 2018 | *I^2^* = 52%, *p* = 0.12 | REM | 0.77 [0.48, 1.25] | 0.29 |
| MA4 Schwartz 2021 | *I^2^* = 44%, *p* = 0.17 | FEM | 0.77 [0.67, 0.89] | < 0.01 |
| MA5 Schwartz’ 2021 | *I^2^* = 63%, *p* = 0.07 | REM | 0.82 [0.47, 1.44] | 0.48 |
| MA6 Steg 2019 | *I^2^* = 52%, *p* = 0.12 | REM | 0.77 [0.47, 1.26] | 0.3 |
| MA7 Goodman 2019 | *I^2^* = 52%, *p* = 0.12 | REM | 0.77 [0.47, 1.25] | 0.29 |


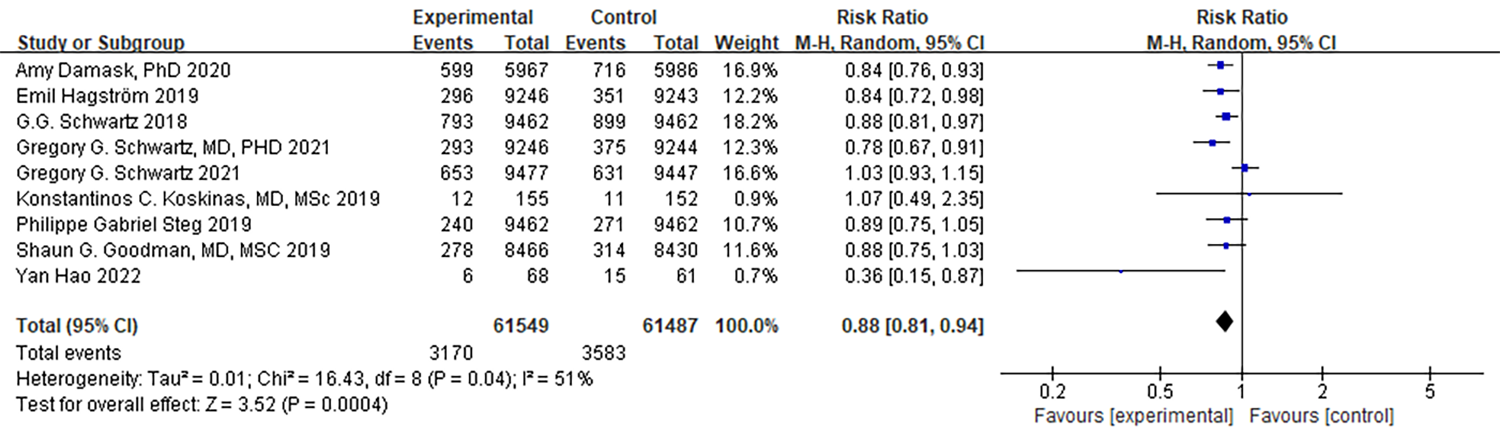


Supplementary Fig. 1. MACE heterogeneity


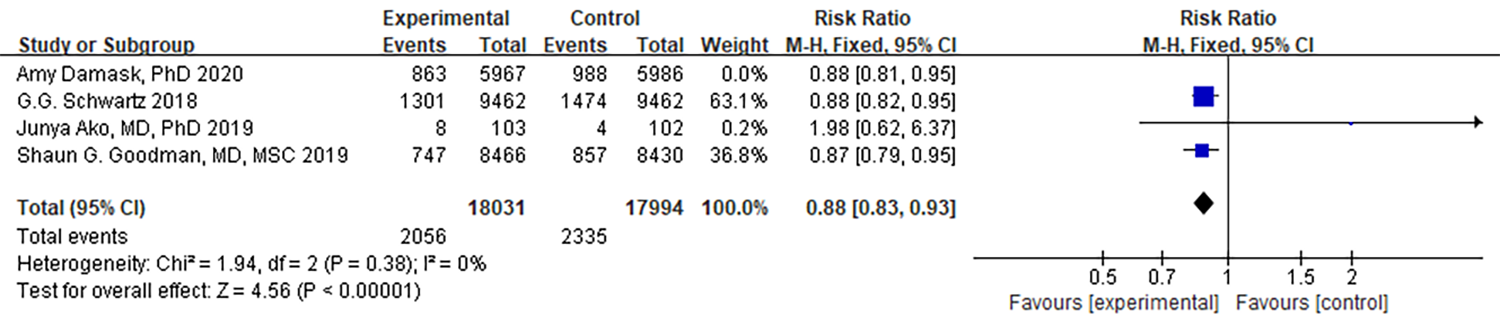


Supplementary Fig. 2. Any ACE heterogeneity


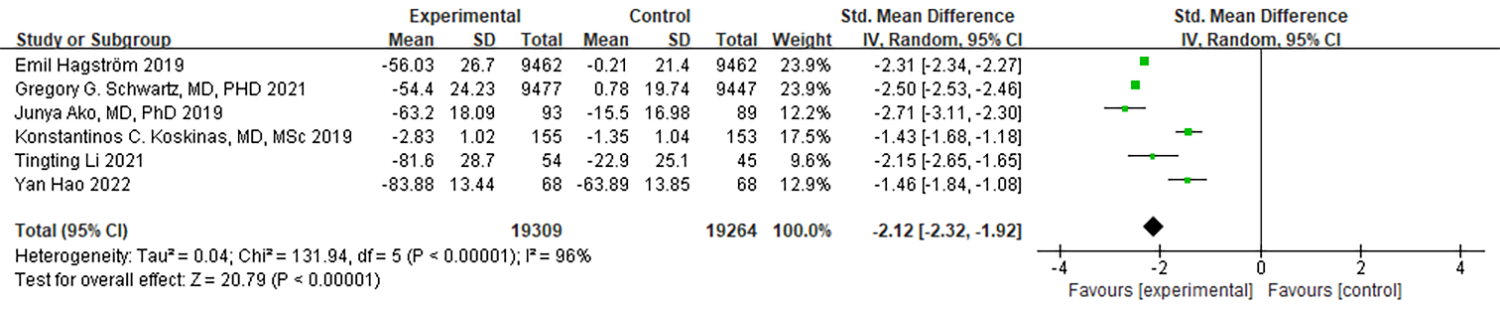


Supplementary Fig. 3. LDL-C heterogeneity and sensitivity analysis


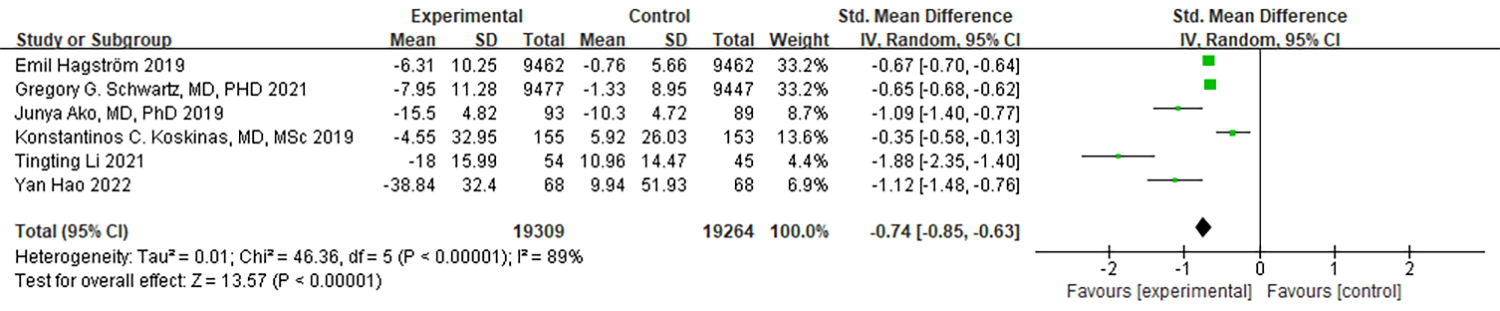


Supplementary Fig. 4. Lipoprotein(a) heterogeneity and sensitivity analysis
